# Protocol for assessing senescence-associated lung pathologies in mice

**DOI:** 10.1016/j.xpro.2021.100993

**Published:** 2021-12-04

**Authors:** Koichiro Kawaguchi, Michihiro Hashimoto, Ryuta Mikawa, Azusa Asai, Tadashi Sato, Masataka Sugimoto

**Affiliations:** 1 Research Institute, National Center for Geriatrics and Gerontology, Obu, Aichi 474-8511, Japan; 2 Division of Tumor Pathology, Department of Pathology, Asahikawa Medical University, Asahikawa 078-8510, Japan; 3 Department of Respiratory Medicine, Juntendo University Graduate School of Medicine, Tokyo 113-8421, Japan; 4 Department of Molecular Aging Research, Nagoya University Graduate School of Medicine, Nagoya 466-8560, Japan; 5 Tokyo Metropolitan Institute of Gerontology, Tokyo 173-0015, Japan

**Keywords:** Cell Biology, Health Sciences, Model Organisms

## Abstract

Cellular senescence underlies tissue aging and aging-associated pathologies, as well as lung pathology. We and others have shown that elimination of senescent cells alleviates pulmonary diseases such as fibrosis and emphysema in animal models. We herein describe a protocol for assessing senescence-dependent lung phenotypes in mice. This protocol describes the use of ARF-DTR mice for semi-genetic elimination of lung senescent cells, followed by a pulmonary function test and the combination with pulmonary disease models to study lung pathologies.

For complete details on the use and execution of this protocol, please refer to Hashimoto et al. (2016), Kawaguchi et al. (2021), and Mikawa et al. (2018).

## Highlights

• Cellular senescence promotes lung aging and diseases
• Detection and elimination of senescent lung cells in ARF-DTR mice
• Assessment of senescence-associated phenotypes in lung tissue

## Graphical abstract

**Figure:**
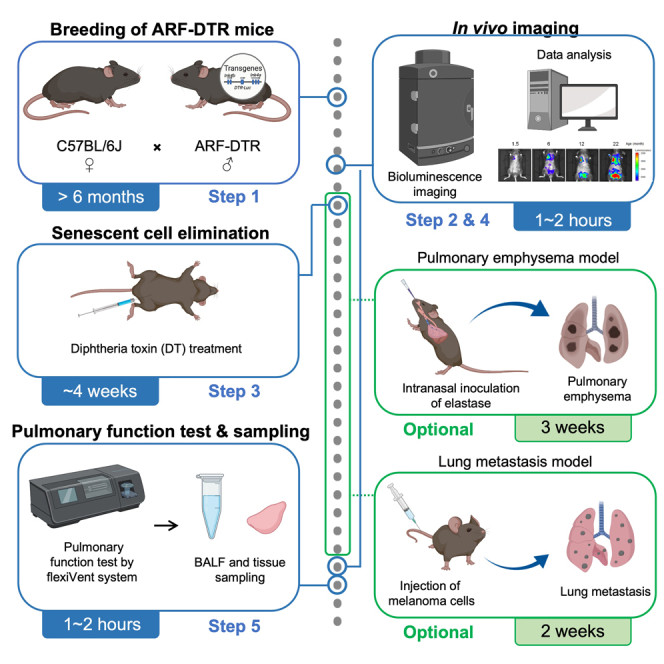

## Before you begin

**Figure 1:**
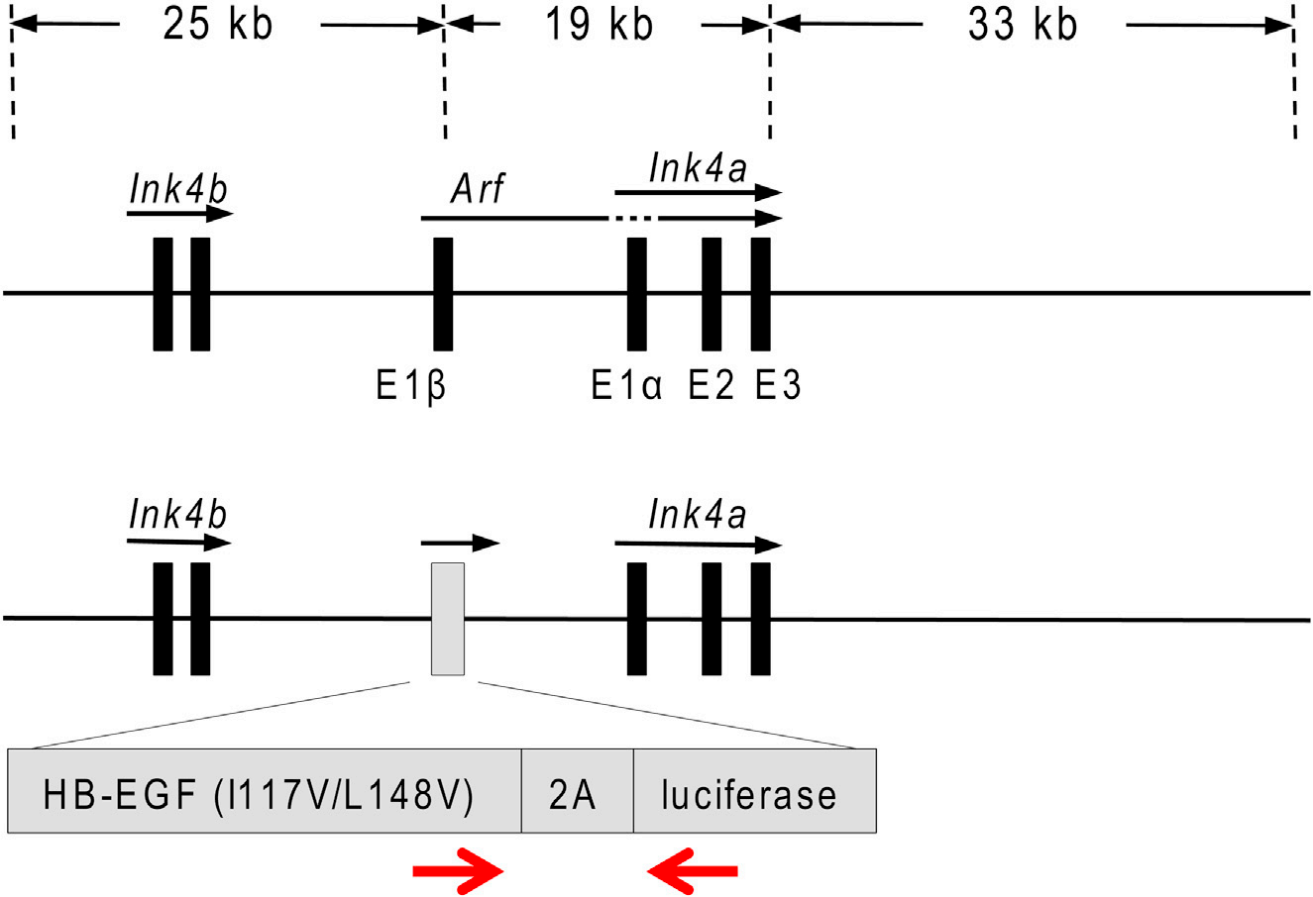
Gene map of ARF-DTR mice A phage artificial chromosome containing the mouse *Ink4a/Arf* locus was used to construct the transgenic vector. Exon 1β of *Arf* was replaced by the gene encoding diphtheria toxin receptor (DTR, human HB-EGF I117V/L148V), which is fused to 2A peptide sequence from self-cleaving picornavirus and firefly luciferase. Red arrows indicate the positions of ARF-DTR genotyping primers. E1β, E1α, E2 and E3 represent exon1β, exon1α, exon2 and exon3 of the *Cdkn2a*, respectively.

The protocol below describes the specific steps for assessing senescence-dependent lung phenotypes in mice. In this protocol, we use a transgenic model, ARF-DTR mice, from which it is possible to eliminate p19^Arf^-expressing cells using diphtheria toxin (DT)-mediated cell knockout system (Furukawa et al., 2006; Saito et al., 2001). p19^Arf^ plays an essential role in the induction of cellular senescence in rodent cells (Kamijo et al., 1997), and its expression increases during aging in the mouse tissues similarly to p16^INK4a^ (Krishnamurthy et al., 2004). ARF-DTR mice have transgene in which *Arf* exon 1β was replaced with genes encoding the diphtheria toxin receptor (DTR) fused to 2A peptide sequence and firefly luciferase (Figure 1). The luminescence signals observed in aged ARF-DTR mice are attributed to those in the lung, adipose, and testis tissues. We successfully eliminated p19^Arf^-expressing cells from the lung tissue of ARF-DTR mice (> 6 months). While we used C57BL/6J background in all of the following analyses, we assume the protocols can also be adapted to Balb/c strain, as this strain has also been reported to show the similar aging-dependent changes in the lung structure and function as well as elastase-induced emphysema pathologies (Kawakami et al., 1984; Limjunyawong et al., 2015).

Institutional permission and oversight information for the animal study should be obtained. All animal experiments in this study were approved by the National Center for Geriatrics and Gerontology Animal Ethics Committee (approval numbers, 2-7, 3-2, 28-6, 29-24, 30-34 and 31-3).

### Breeding animals

**Timing: >6 months prior to experiment**

1. Breeding ARF-DTR mice
  a. Hemizygous ARF-DTR transgenic mice (Hashimoto et al., 2016) are crossed with C57BL/6J mice to obtain transgene^+^ and transgene^-^ littermate animals.

***Note:*** Male ARF-DTR mice and female wild-type mice are typically used for breeding so that ARF-DTR hemizygous offspring can efficiently be obtained.

**Figure 2:**
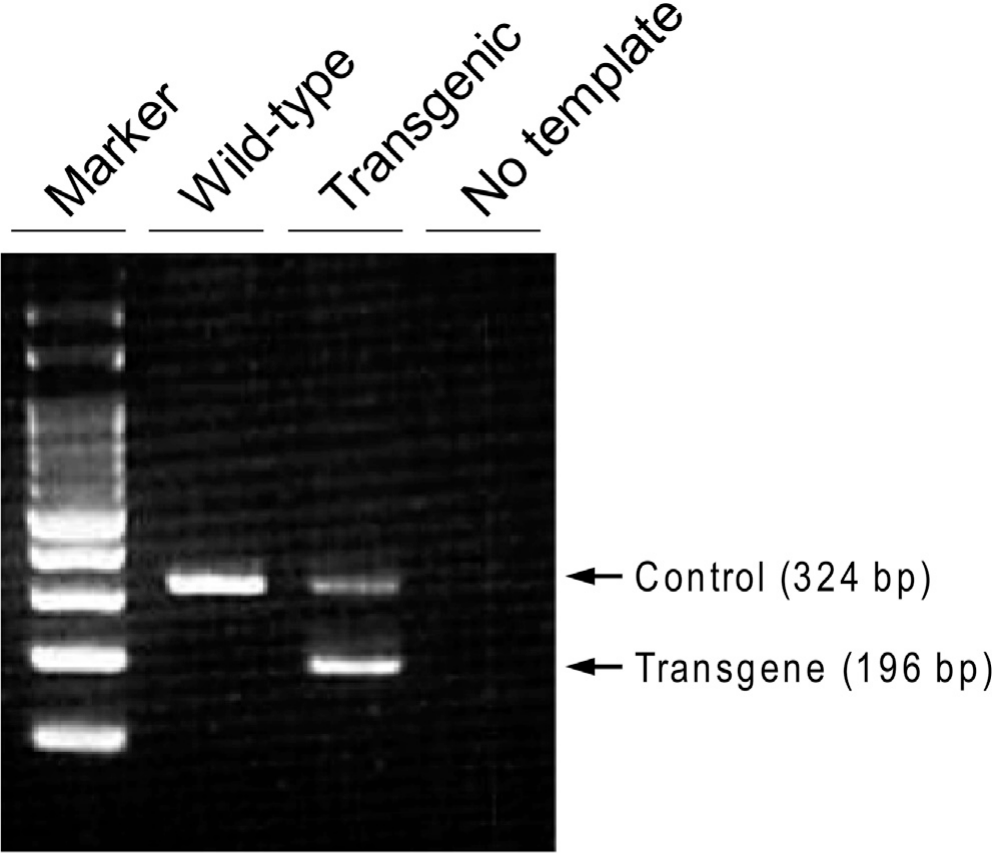
Genotyping of ARF-DTR mice PCR products were separated by 2% agarose gel electrophoresis using a Mupid-exU electrophoresis system and visualized by ethidium bromide staining. Four μL of PCR products were loaded.

2. Genotyping
  a. Cut the tails (5 mm) of pups and put in a 1.5 mL tube.
  b. Tail lysis
    i. Add 500 μL of tail lysis buffer and 2 μL Proteinase K.
    ii. Incubate overnight (6–12 h) at 55°C.
    iii. Vortex thoroughly, and check that the tail is completely lysed.
  c. Phenol/chloroform extraction of genomic DNA
    i. Add 500 μL of neutralized phenol/chloroform and vortex thoroughly.
    ii. Centrifuge at 15,300 × *g* for 5 min at room temperature (18°C–25°C).
    iii. Transfer the upper phase to a new 1.5 mL tube.
    iv. Add 350 μL of isopropanol and invert until DNA precipitate forms.
    v. Centrifuge at 15,300 × *g* for 5 min at room temperature and carefully remove and discard supernatant.
    vi. Add 0.5–1 mL of 70% ethanol and invert several times.
    vii. Centrifuge at 15,300 × *g* for 5 min at room temperature and carefully remove and discard supernatant.
    viii. Air dry at room temperature.
    ix. Add 200 μL of H_2_O and dissolve the DNA pellet.
  d. Use 20–50 ng of genomic DNA for genotyping PCR.
    i. Perform multiplex PCR to amplify the transgene (DTR-Luc) and control allele using the primers described in the key resources table (KRT). PCR condition is described in materials and equipment.
      ***Note:*** This genotyping PCR protocol is designed for the use of KOD One® PCR Master Mix (TOYOBO) and a T100™ thermal cycler (Bio-Rad).
    ii. Separate the PCR products by agarose gel electrophoresis. The amplicon sizes of control allele and transgene are 324 bp and 196 bp, respectively (Figure 2).

## Key resources table

**Table:** 

REAGENT or RESOURCE	SOURCE	IDENTIFIER
**Chemicals, peptides, and recombinant proteins**		

Proteinase K	Sigma-Aldrich	Cat#P4850
Trisaminomethane (Tris)	FUJIFILM Wako Pure Chemical	Cat#201-06273
Sodium dodecyl sulfate (SDS)	FUJIFILM Wako Pure Chemical	Cat#194-13985
Phosphate-buffered saline (PBS)	FUJIFILM Wako Pure Chemical	Cat#162-19321
Phenol/chloroform	FUJIFILM Wako Pure Chemical	Cat#311-90151
Isopropanol (2-Propanol)	FUJIFILM Wako Pure Chemical	Cat#166-04836
KOD One® PCR Master Mix -Blue-	TOYOBO	Cat#KMM-201
Pentobarbital sodium	Kyoritsu Seiyaku	Somnopentyl
Mildform®20N	FUJIFILM Wako Pure Chemical	Cat#136-10041
Diphtheria toxin	Sigma-Aldrich	Cat#D0564
CELLBANKER® 1 plus	ZENOGEN PHARMA	Cat#CB021
RNAlater®	Thermo Fisher Scientific	Cat#AM7021
TRI reagent®	Molecular Research Center	Cat#TR118
VivoGlo™ Luciferin, in vivo grade	Promega	Cat#P1043
Elastase, from porcine pancreas	Elastin Products Company	Cat#EC134
DMEM high glucose	Sigma-Aldrich	Cat#D5796
FBS	Biofill	Cat#FBS01-500
0.25w/v% Trypsin-1mmol/L EDTA·4Na Solution with Phenol Red	FUJIFILM Wako Pure Chemical	Cat#209-16941
Penicillin-Streptomycin (×100)	FUJIFILM Wako Pure Chemical	Cat#168-23191

**Experimental models: Cell lines**		

B16-F10	ATCC	CRL-6475

**Experimental models: Organisms/strains**		

Mouse: ARF-DTR	Hashimoto et al. (2016)	n/a
Mouse: C57BL/6J	SLC	C57BL/6JJmsSlc

**Oligonucleotides**		

ARF-DTR genotyping Transgene, forward: TTTAGGTACCATAGGAGAGGAGG	Integrated DNA Technologies	n/a
ARF-DTR genotypingTransgene, reverse: CATCTTCCAGCGGATAGAATGGC	Integrated DNA Technologies	n/a
ARF-DTR genotyping Control allele, forward: CTAGGCCACAGAATTGAAAGATCT	Integrated DNA Technologies (Sequence is derived from the Jackson Laboratory protocol, internal positive control primer oIMR7338)	n/a
ARF-DTR genotyping Control allele, reverse: GTAGGTGGAAATTCTAGCATCATCC	Integrated DNA Technologies (Sequence is derived from the Jackson Laboratory protocol, internal positive control primer oIMR7339)	n/a

**Software and algorithms**		

Living Image 3.0	PerkinElmer	n/a
flexiVent software	SCIREQ	n/a

**Other**		

Mupid® -exU	Mupid	Cat#EXU-1
IVIS imaging system	PerkinElmer	n/a
Forceps	Fine Science Tools	Cat#11049-10
flexiVent system	SCIREQ	n/a
Silicon tube	SAINT-GOBAIN	Cat#ACFJ00002
Manometer (testo 510)	Testo	Cat#0563 0510
T-shaped (3-way) stopcock	TERUMO	Cat#TS-TR2K
T100™ thermal cycler	Bio-Rad	Cat#1861096J1
Myjector® syringe (27G × 1/2")	TERUMO	Cat#SS-10M2713A

## Materials and equipment

**Table:** Tail lysis buffer

Reagent	Final concentration	Amount
1M Tris-HCl (pH 7.4)	50 mM	25 mL
0.25M EDTA (pH8.0)	100 mM	200 mL
5M NaCl	100 mM	10 mL
10% SDS	1%	50 mL
ddH_2_O	n/a	to 500 mL

Filtrate and store the buffer at room temperature. The buffer can be stored for up to 1 year.

**Table:** ARF-DTR mice genotyping PCR

PCR cycling conditions			
Steps	Temperature	Time	Cycles
Initial Denaturation	94°C	3 min	1
Denaturation	94°C	30 s	32 cycles
Annealing	62°C	1 min	
Extension	72°C	1 min	
Final extension	72°C	2 min	1
Hold	4°C	store

**Table:** Luciferin stock solution

Reagent	Final concentration	Amount
VivoGlo Luciferin, in vivo grade	30 mg/mL	1 g
ddH_2_O	n/a	33.3 mL

Aliquot stock solution into 600 μL/tube and store at −80°C for up to 1 year. Avoid repeated freeze-thawing.

**Table:** Luciferin solution for injection

Reagent	Final concentration	Amount
Luciferin stock solution (30 mg/mL)	15 mg/mL	600 μL
PBS	n/a	600 μL

Prepare the solution just before use. Injection volume: Body weight × 10 μL/mouse (150 mg/kg)

**Table:** DT stock solution

Reagent	Final concentration	Amount
Diphtheria toxin	1 mg/mL	1 mg
ddH_2_O	n/a	1 mL

Aliquot stock solution into 10 μL/tube and store at −80°C for up to 1 year. Avoid freeze-thawing.

**Table:** Injectable DT solution

Reagent	Final concentration	Amount
DT stock solution (1 mg/mL)	5 μg/mL	5 μL
PBS	n/a	995 μL

Prepare the solution just before use. Injection volume: Body weight × 10 μL/mouse (50 μg/kg)

**Table:** B16-F10 cells culture medium

Reagent	Final concentration	Amount
DMEM high glucose	n/a	445 mL
FBS	10 %	50 mL
Penicillin-Streptomycin (×100)	× 1	5 mL

Store at 4°C, and do not store more than 3 months.

## Step-by-step method details

### *In vivo* imaging

**Timing: 1**–**2 h**

This step describes how to monitor senescent cells by *in vivo* imaging.

1. Prior to *in vivo* imaging, shave hair on the ventral side with an electric clipper (Figure 3A).

**Figure 3:**
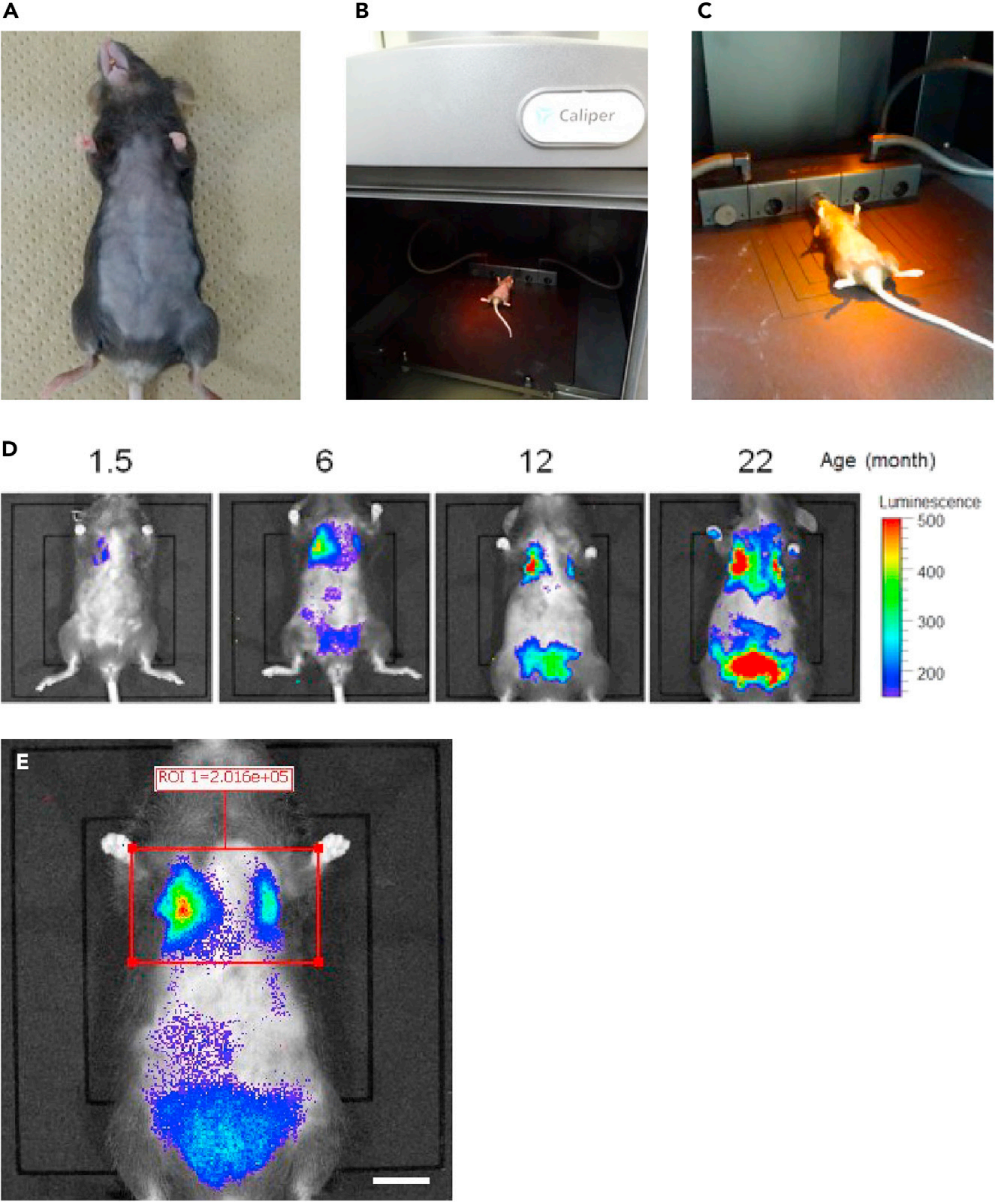
*In vivo* imaging analysis of ARF-DTR mice (A) Photo of the mouse shaved hair on the ventral side. (B and C) Photos showing the mouse inside the IVIS spectrum imager. (D) Luciferase activity was observed in the lung and adipose tissue with aging in female ARF-DTR mice. (E) Photo of drawing the ROI by Living image software. Place a rectangle over the area of interest. Scale bar; 10 mm.
2. Set up imaging settings at the IVIS spectrum imager system as follows; Exposure time: 3 min, Binning: Medium, F/Stop: 1, Emission Filter: Open, Field of View: B.
3. *In vivo* imaging
  a. Inject the luciferin (VivoGlo^TM^ Luciferin, *in vivo* grade) intraperitoneally (i.p.) according to the manufacturer’s instructions (https://www.promega.jp/products/luciferase-assays/reporter-assays/vivoglo-luciferin-in-vivo-grade/?catNum=P1043#resources).
  b. Anesthetize mice with 2% isoflurane in air.
  c. Place the mice in the chamber and arrange them as necessary so that you can begin imaging (Figures 3B and 3C).
  d. Perform the bioluminescence imaging 10 min post i.p. injection (Figure 3D). Troubleshooting 1

**CRITICAL:** Because dark colored hair impairs the luminescence detection, hair on the ventral side should be removed thoroughly before *in vivo* imaging.

***Note:*** In ARF-DTR mice, luminescence reflecting senescent cells in the tissue is mainly obtained from lung, adipose tissue and testis.

4. To calculate light outputs, draw region of Interest (ROI) by Living image software (Figure 3E).

### Senescent cell elimination

**Timing: ∼4 weeks**

**Figure 4:**
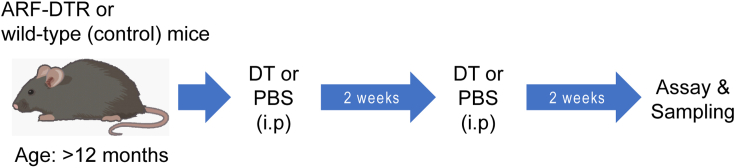
Experimental scheme for senescent cell elimination in ARF-DTR mice

This step describes how to eliminate senescent cells from lung tissue (Hashimoto et al., 2016). The experimental scheme for senescent cell elimination is described in Figure 4.

5. DT treatment
  a. Freshly prepare the DT solutions (5 μg/mL in PBS) and keep them on ice until use.
  b. Inject DT solution (50 μg/kg body weight) intraperitoneally twice with a 2-week interval.

**CRITICAL:** Do not use the refrozen DT solution.

6. Four weeks after DT injection, evaluate senescent cell elimination by *in vivo* imaging described in the steps 1–3. Troubleshooting 2

***Note:*** In addition to *in vivo* imaging, we recommend assessing senescent cell elimination by gene expression analysis of senescent markers such as *Ink4a*, *Arf* and *p21* by RT-qPCR.

### Pulmonary function test

**Timing: ∼1 h**

This step describes how to perform a pulmonary function test by the flexiVent® system.

**Figure 5:**
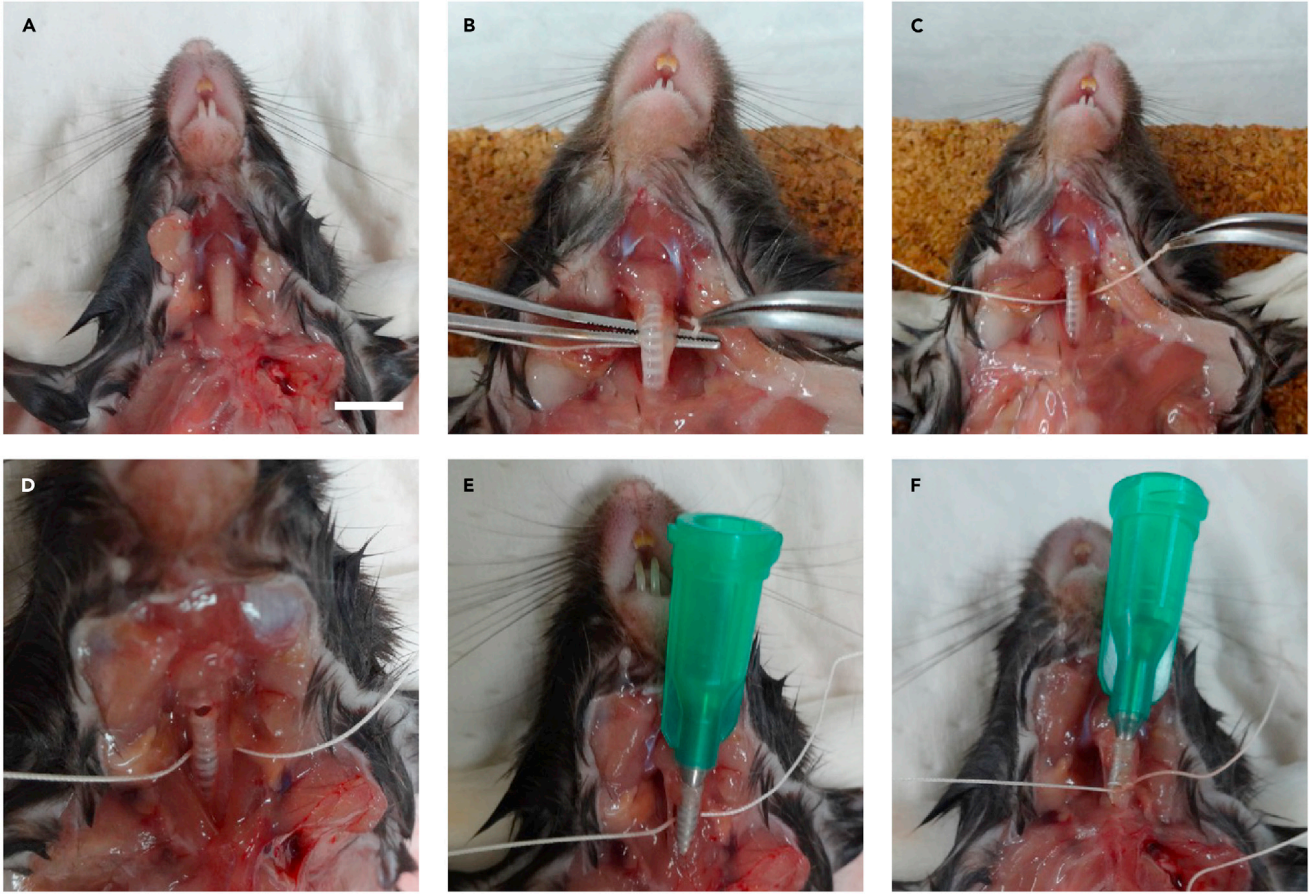
Mouse lung intubation (A) Exposing the trachea for intubation. Scale bar; 5 mm. (B and C) Insert string under the trachea using small forceps. (D) Tracheostomy. Making a small incision into the trachea. (E) Tracheal intubation using a 12 mm-long cannula. (F) Tie off the outside of the intubated site to prevent the cannula from falling out.
9. Insert string under the trachea using small forceps (Figures 5B and 5C).
10. Make a small incision into the trachea (Figure 5D).
11. Perform tracheal intubation using a 12 mm-long cannula (Figure 5E).
12. Tie off the outside of the intubated site to prevent the cannula from falling out (Figure 5F).
13. Connect the intubated cannula to a flexiVent® system.

***Note:*** Diaphragm may be removed before connecting the flexiVent® system to allow visual confirmation of the airflow into the lung tissue.

7. Euthanize mice by intraperitoneal injection of excess amount (100 mg/kg body weight) of pentobarbital sodium.
8. Cut and open the skin to expose the trachea (Figure 5A).

14. Ventilate the mouse at a respiratory rate of 150 breaths/min with a tidal volume of 10 mL/kg against a positive end expiratory pressure of 3 cmH_2_O.
15. Consecutively perform Deep inflation, Snapshot-150, Quickprime-3, and a pressure-volume loop with constant increasing pressure three times in each mouse.
16. Acquire parameters using a flexiVent software.
  a. Calculate the dynamic compliance and resistance values using a single frequency forced oscillation technique.
  b. Calculate the static lung compliance value by fitting the Salazar-Knowles equation to the pressure volume loop.
  c. Obtain the tissue elastance and tissue damping values from respiratory system impedance data using a constant phase model.

### BALF and lung tissue sampling

**Timing: ∼1 h**

This step describes how to obtain bronchoalveolar lavage fluid (BALF) and lung tissue.

**Figure 6:**
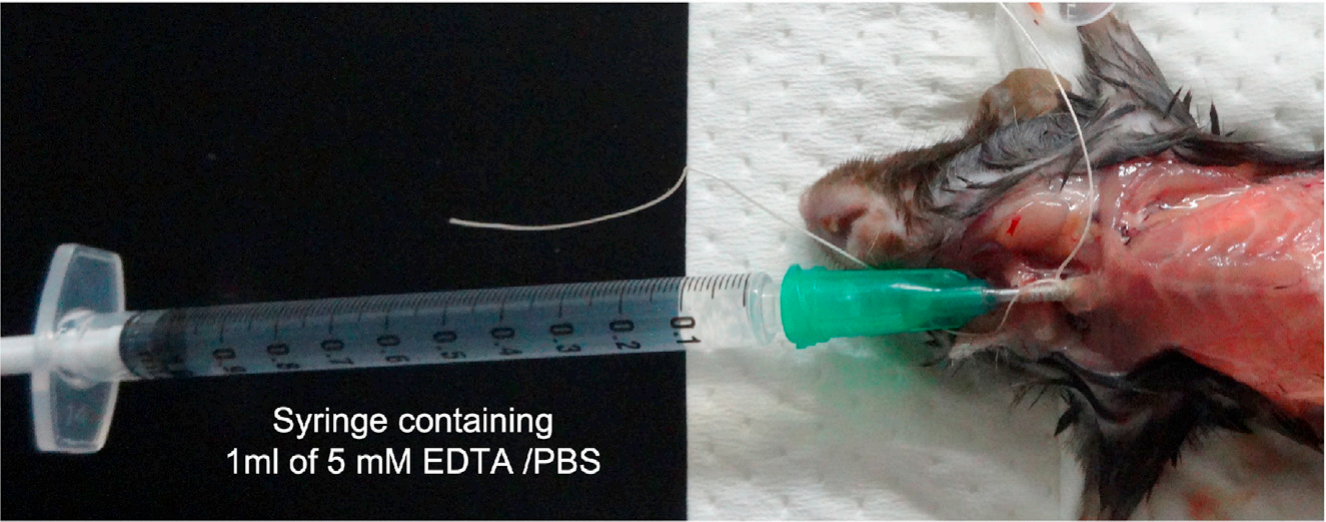
A method for BALF sampling A syringe containing 1 mL of 5 mM EDTA in PBS is connected to the intubated cannula.
18. Bronchoalveolar lavage fluid (BALF) sampling
  a. Gentle syringing for 3 times.
  b. Transfer the BALF to a 1.5 mL tube.
  c. Centrifuge at 5,800 × *g* for 1 min at 4°C.
  d. Transfer the supernatant (BALF) to a new 1.5 mL tube, and store at -80°C until use. Troubleshooting 3
  e. (if needed) Add 100 μL of CELLBANKER® to precipitate (cell fraction), and store at -80°C until use.

***Note:*** For longer storage of BALF cells, store the samples in liquid nitrogen.

**Figure 7:**
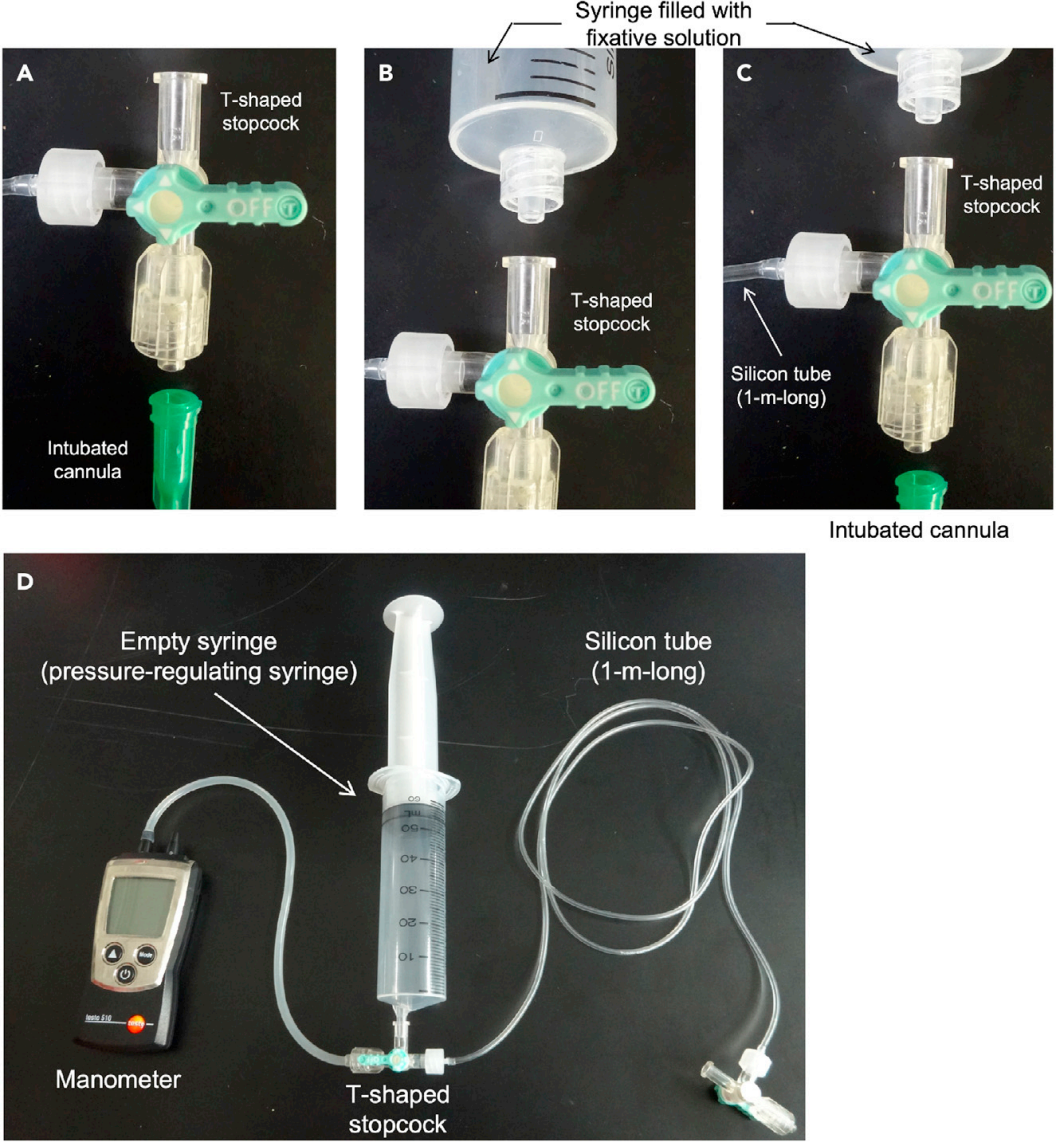
Preparation for lung fixation (A) After the BALF sampling, remove the syringe and connect a T-shaped stopcock to the intubated cannula. (B) Connect a syringe filled with fixative solution to one side of the T-shaped stopcock. (C and D) The other side is connected to a 1-m-long silicon tube which is connected to a manometer and empty syringe (pressure-regulating syringe) through another T-shaped stopcock.
  b. Connect a syringe filled with fixative solution (Mildform®20N) to one side of the T-shaped stopcock (Figure 7B).
  c. The other side is connected to a 1-m-long silicon tube (Figure 7C) which is connected to a manometer and empty syringe (pressure-regulating syringe) through another T-shaped stopcock (Figure 7D).

***Note:*** In case of both RNA/protein extraction and tissue fixations are required from a single mouse, add the next step (step 20) before fixation.

**Figure 8:**
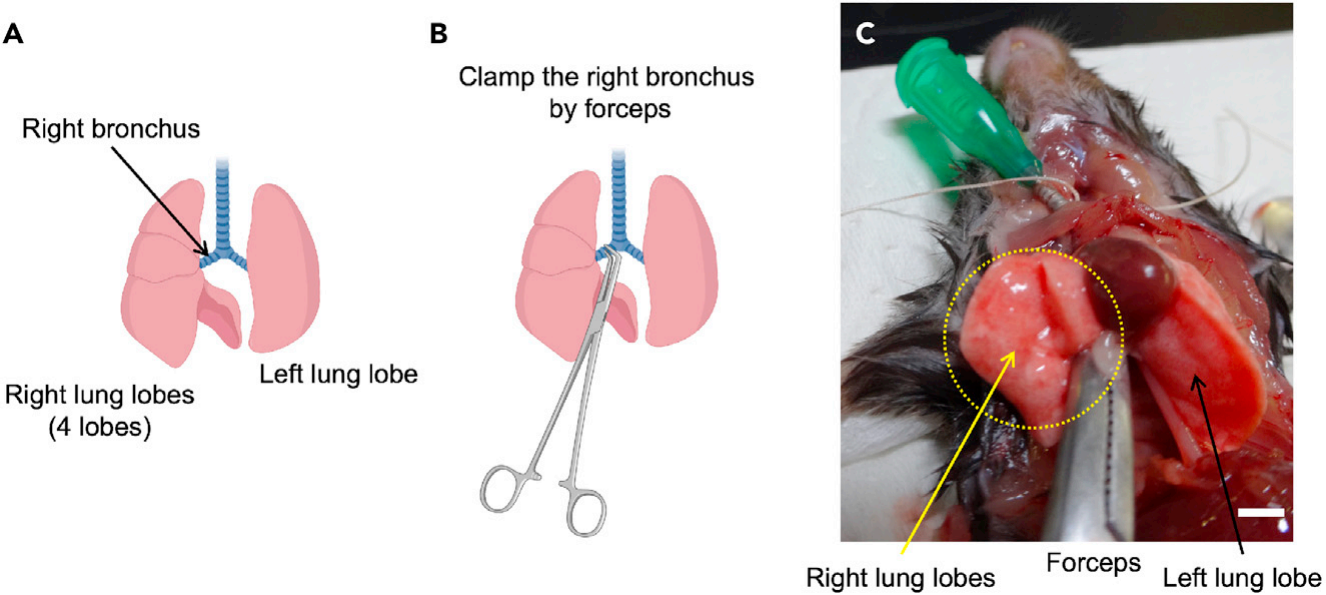
Images of clamping the right bronchus (A) Illustration of mouse lung structure. (B) Illustration of clamping site (right bronchus) with forceps. (C) Photo of clamping the right bronchus with forceps. Scale bar; 5 mm.
  b. Excise the right lung lobes (4 lobes) for RNA extraction.
  c. Wash the right lung lobes twice with 10 mL of PBS.
  d. Mince the tissue into small pieces with sterile scissors in a 2 mL round-bottom tube (Figures 9A and 9B).

17. After pulmonary function test, connect a syringe containing 1 mL of 5 mM EDTA in PBS to the intubated cannula (Figure 6).

19. Preparation for lung fixation
  a. After the BALF sampling, remove the syringe and connect a T-shaped stopcock to the intubated cannula (Figure 7A).

20. Collecting the right lung lobes for RNA/protein extraction
  a. Clamp the right bronchus with forceps (Figures 8A–8C).

**Figure 9:**
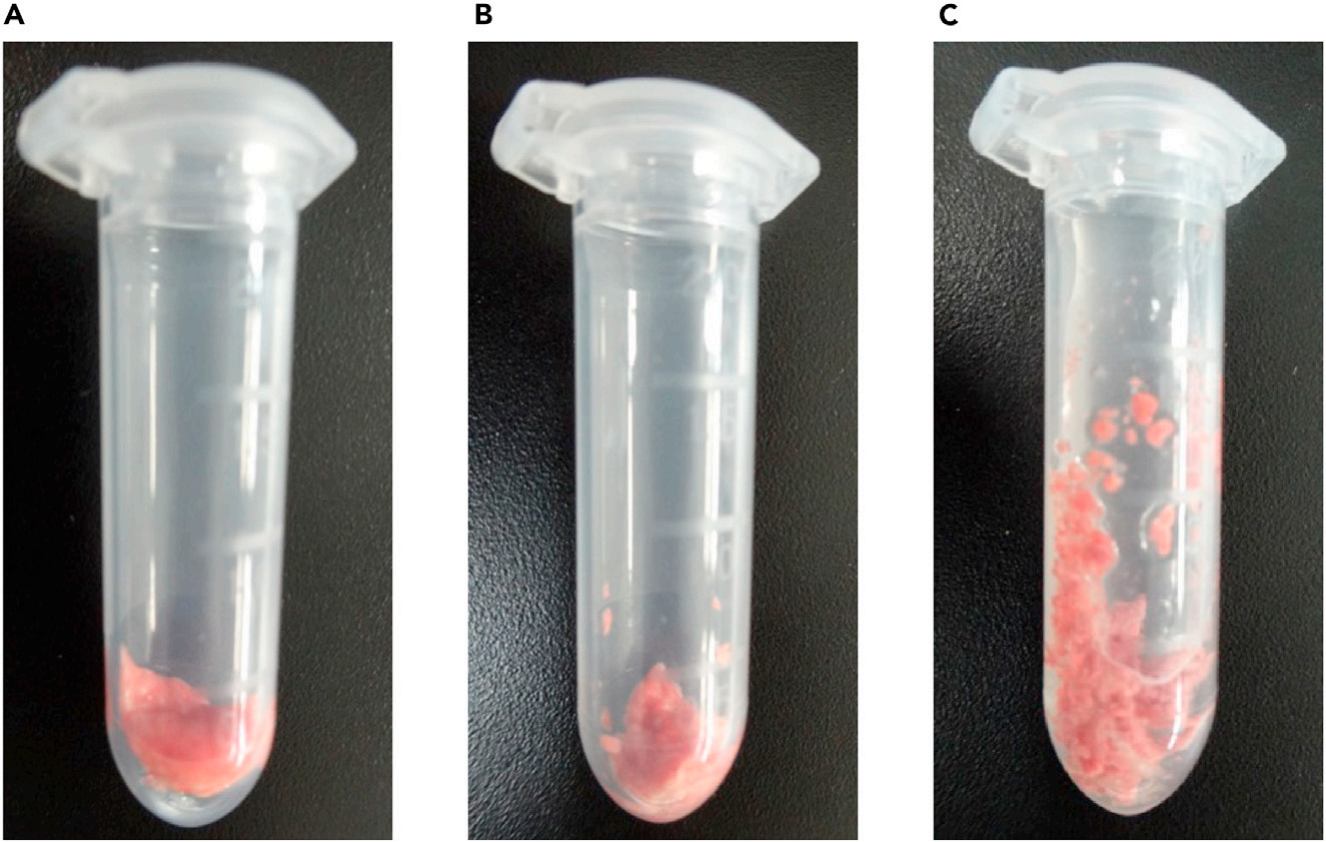
Images of mincing the lung tissue (A) Right lung lobes before mince. (B) Right lung lobes after mince. (C) Minced tissues are soaked in RNAlater®.
  e. For RNA extraction, soak the tissue into RNAlater® and store at -30°C until use (Figure 9C).
  f. For protein extraction, snap freezing by liquid nitrogen and store at -80°C until use.
  g. RNA/protein extraction using TRI REAGENT®. Troubleshooting 4

**Figure 10:**
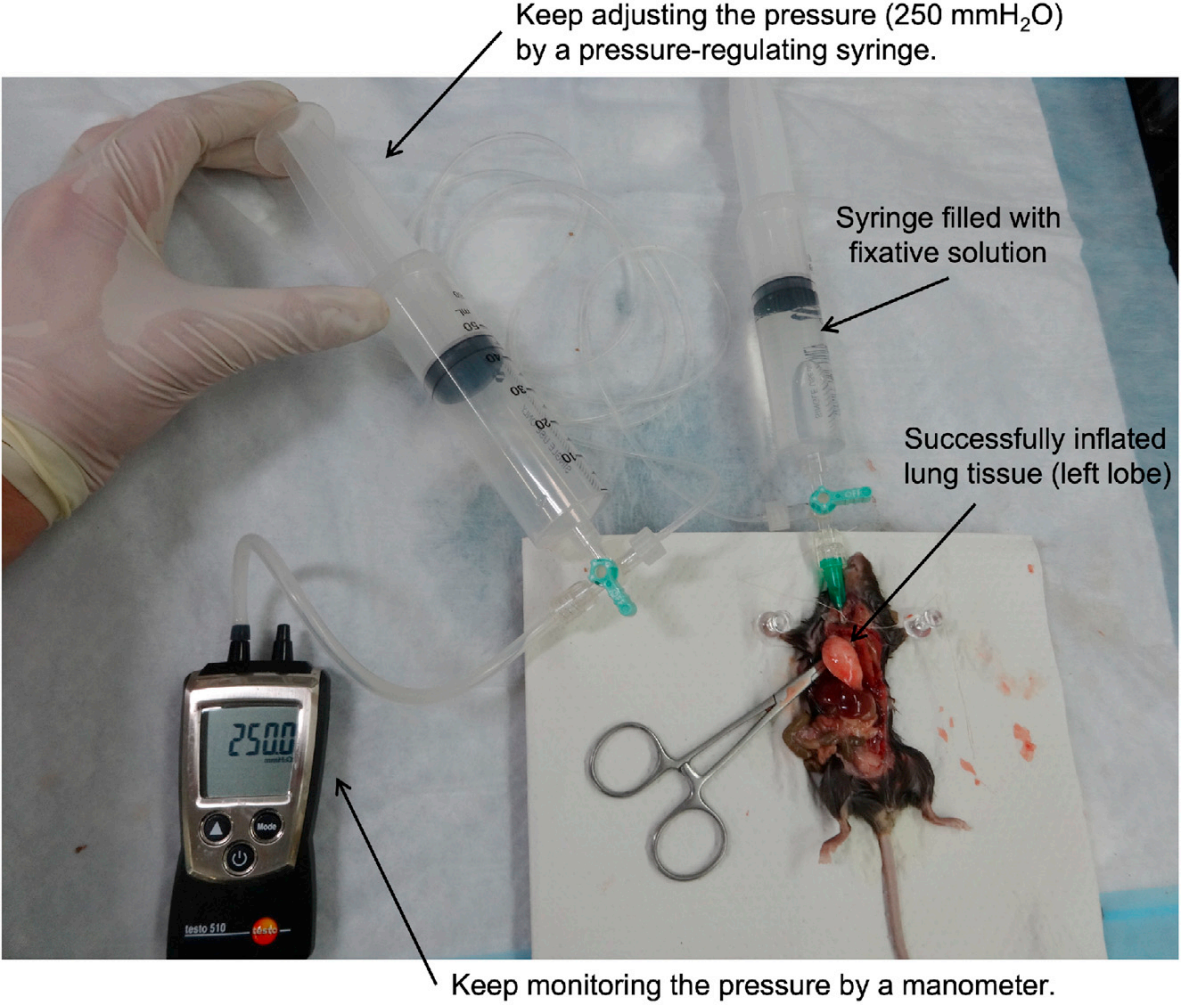
Handling of pressure-regulating syringe After inflating lung tissue by injecting the fixative solution, keep monitoring and adjusting the pressure by manometer and pressure-regulating syringe during fixation.

**Figure 11:**
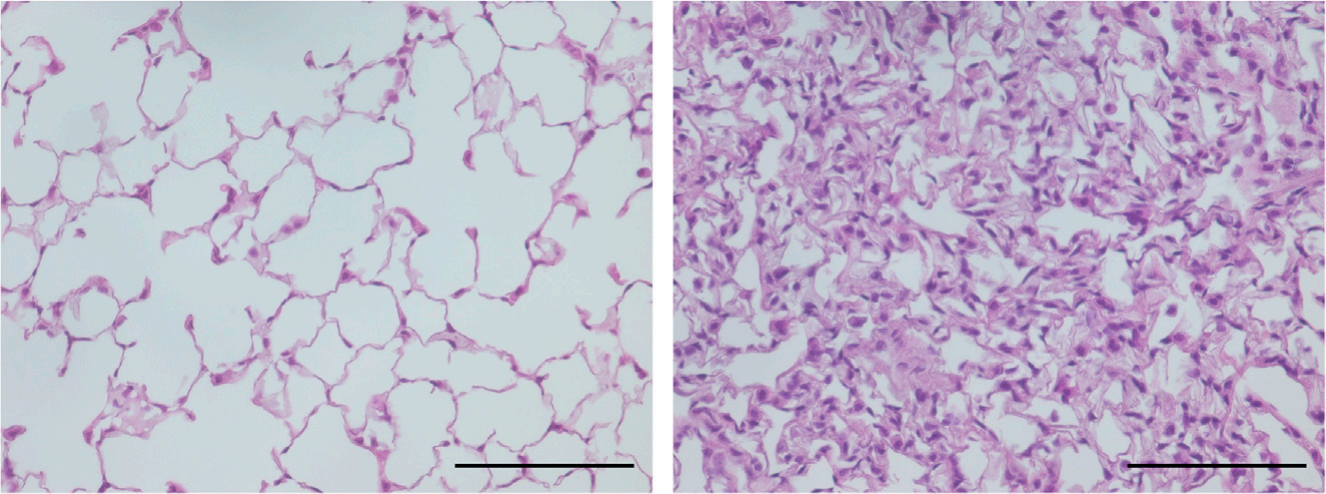
Inflated lung tissue Sections (5 μm thickness) of inflated (*left*) or uninflated lung tissue (*right*) were stained with hematoxylin and eosin. Representative images were shown. Bar; 60 μm.

21. Fix the lung tissue.
  a. Inflate lung tissue by injecting the fixative solution at constant pressure (250 mmH_2_O) for 10 min (Figure 10).
    **CRITICAL:** Keep monitoring the manometer to maintain the pressure using the pressure-regulating syringe during fixation (Figure 10).
  b. Excise lung tissue (left lung lobe if using step 20) and store it in fixative solution until use. Tissue is fixed at least 1 day and used within 3 days. For longer storage, replace fixative solution with 70% ethanol.

22. Assess lung phenotypes arise from senescent cells’ elimination by morphometric analysis and immunohistochemistry (Figure 11). Troubleshooting 5

### Pulmonary emphysema model

**Timing: 3 weeks**

**Figure 12:**
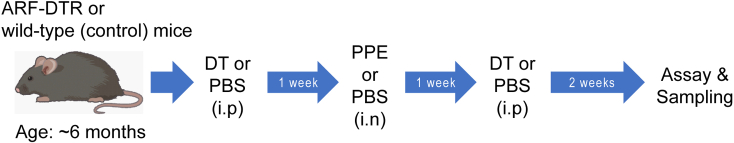
Experimental design to analyze the impacts of senescent cell elimination in elastase-induced pulmonary emphysema model

This step describes how to assess senescence-dependent lung pathologies by combining with the pulmonary emphysema model using porcine pancreatic elastase (Mikawa et al., 2018). The experimental scheme for setting up pulmonary emphysema model is described in Figure 12.

23. Intranasally administrate 100 μL of porcine pancreatic elastase (PPE, 5 units /100 μL PBS).

***Note:*** PPE administration should be done one week after first DT treatment.

24. One week after PPE administration, inject DT solution (50 μg/kg body weight) intraperitoneally (second DT treatment).
25. Two weeks after DT injection, perform the analyses described in the steps 7–22 (pulmonary function test, BALF isolation and tissue sampling).

### Lung metastasis model

**Timing: 2 weeks**

**Figure 13:**
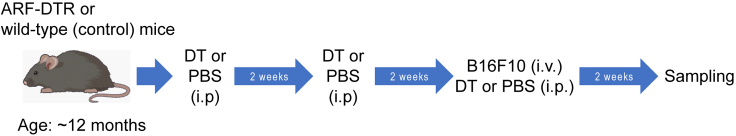
Experimental design to analyze the effects of senescent cell elimination in melanoma lung metastasis model

This step describes how to assess senescence-dependent lung pathologies by combining with B16-F10 melanoma lung metastasis model (Kawaguchi et al., 2021). The experimental scheme for setting up lung metastasis model is described in Figure 13.

26. Culture of B16-F10 cells.
  a. Prepare complete growth medium (DMEM supplemented with 10% fetal bovine serum, 100 units/mL penicillin and 100 μg/mL streptomycin).
  b. Seed B16-F10 cells at 0.5 to 2 × 10^6^ cells/T75 flask containing 15 mL of complete growth medium.

***Note:*** Replace medium every 2–3 days, and a subcultivation ratio of 1:10 is recommended.

***Note:*** Incubate the cells in a CO_2_ incubator (37°C, 5% CO_2_) for 2–4 days so that the cells are approximately 80–90% confluent on the day of injection (2–3 days with seeding density at 2.6 × 10^4^ cells/cm^2^, and 3–4 days with seeding density at 6.6 × 10^3^ cells/cm^2^).

27. Prepare cell suspension for tail vein injection.
  a. Remove and discard culture media from flask.
  b. Briefly rinse the cell layer with PBS.
  c. Add 2 mL of trypsin-EDTA solution to the flask.
  d. Incubate the cells in a CO_2_ incubator (37°C, 5% CO_2_) until the cell layer is dispersed (usually within 2 min).
  e. Add 8 mL of complete growth medium and aspirate cells by gently pipetting.
  f. Transfer cells to a 50 mL tube and perform cell counting.
  g. Centrifuge at 400 × *g* for 3 min at room temperature.
  h. Remove and discard the supernatant.
  i. Add 10 mL of PBS and wash cells by gently pipetting.
  j. Centrifuge at 400 × *g* for 3 min at room temperature.
  k. Remove and discard the supernatant.
  l. Repeat steps i-k.
  m. Resuspend cells in PBS at a density of 2 × 10^6^ cells/mL.
28. Inject 200 μL of B16-F10 cell suspension (4 × 10^5^ cells /mouse) into the tail vein.

***Note:*** B16-F10 injection should be done after DT treatment (twice with a 2-week interval).

***Note:*** We use Myjector® syringe (27G × 1/2", TERUMO) for tail vein injection.

29. DT treatment
  a. Freshly prepare the DT solutions (5 μg/mL in PBS) and keep them on ice until use.
  b. Inject DT solution (50 μg/kg body weight) intraperitoneally.
30. Two weeks after B16-F10 injection, euthanize mice and collect lung tissue.
31. Dissect lung tissue and count the number of metastatic nodules under a stereomicroscope.

***Note:*** When lung tissue is also required for other analysis (e.g., IHC), use the left lung lobe for metastasis site counting.

## Expected outcomes

**Figure 14:**
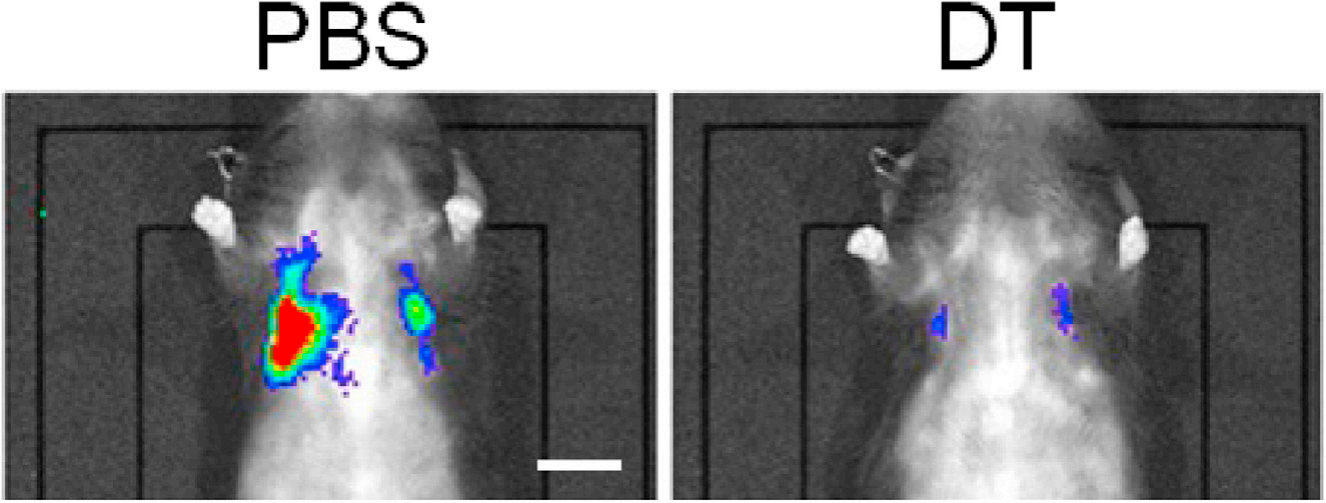
Elimination of p19^Arf^-expressing cells from lung tissue Twelve-month-old ARF-DTR mice were intraperitoneally injected with PBS (left) or DT (right). Luciferase activity was monitored by *in vivo* imaging 2 days after the injection. Scale bar; 10 mm.

The below data (Figure 14) represent expected results of lung phenotype by senescent cell elimination from ARF-DTR mice. Luciferase activity was detected in the lung area of the PBS-treated ARF-DTR mouse (*left*), which was hardly detectable in the DT-treated animal (*right*), suggesting that lung senescent cells expressing the transgene (luciferase and DT) were eliminated by DT.

## Limitations

While the toxin receptor-mediated cell knockout system is a powerful tool to specifically eliminate senescent cells from tissues, there are some points that should be noted for the use of DT. First, continuous DT administration might produce anti-DT antibodies in the mouse body, which might neutralize the administered DT and prevent the elimination of senescent cells from tissues (Kimura et al., 2007). Thus, prolonged DT administration should be avoided. Second, in principle, DT should not work in mice without DTR, but frequent administration of DT shows toxicity probably due to the off-target effects. We thus recommend administrating DT with at least a 2-week interval. Elimination of senescent cells can be confirmed by *in vivo* imaging two days after DT injection. However, at least 2 weeks will be needed to assess the biological effect of senescent cell depletion in lung tissue.

In ARF-DTR mice, bioluminescence images can be mainly obtained in the lung and adipose tissue with aging. However, the expression level of ARF is increased in many other tissues during aging, suggesting that transgene expression in ARF-DTR mice does not fully mimic the expression pattern of the endogenous *Arf*. In male ARF-DTR mice, bioluminescence can be also obtained from testis regardless of aging and is decreased by DT treatment, indicating elimination of p19^Arf^-expressing cells from testis. While no difference between male and female has been observed in the above lung experiments, results should be carefully interpreted when ARF-DTR mice are utilized for other types of analysis.

While the presence of senescent cells in lung tissue can be confirmed as early as 6 months old by *in vivo* imaging, respiratory function has not yet declined at that time. Therefore, to evaluate age-dependent changes in lung function, it is recommended to use ARF-DTR mice older than 12 months of age. Disease models (e.g., emphysema model) will be needed to see the effects of senescent cell elimination in younger animals.

## Troubleshooting

### Problem 1

No or very weak bioluminescence detected during *in vivo* imaging (step 3).

### Potential solution

This could be mostly caused by incomplete substrate injection. Repeat the luciferin injection to solve the problem.

Additionally, dark colored hair highly affects optical imaging by blocking and absorbing the photon. It is essential to shave the hair thoroughly around the area to observe when using the black mice. Alternatively, the use of white hair background is recommended.

### Problem 2

Failure in senescent cells elimination (steps 5 and 6)

### Potential solution

In most cases the possible reason is losing bioactivity of DT. The freshly prepared DT solution should be used at all times. Do not use the refrozen DT solution, because DT is very sensitive to freeze-thawing.

### Problem 3

Blood contamination in the BALF (step 18)

### Potential solution

Carefully cut and open the skin to expose the trachea (step 8). If blood vessels are damaged and bleeding occurs, flush with PBS before tracheostomy (step 9).

### Problem 4

Low RNA yield and quality (step 20)

### Potential solution

Low yield and quality may be caused by excess amounts of tissue. To improve the yield and quality, reduce the amount of starting material. Homogenize tissue samples in 1 mL of TRI reagent per 50–100 mg of tissue. More than 1 μg per mg of lung tissue with OD260/OD280 >1.9 is expected.

### Problem 5

Unclear lung structure from the tissue section (step 22).

### Potential solution

In most cases the possible reason is insufficient lung inflation. This could be caused by a leakage of fixative solution from the clamped site of the right bronchus. To avoid the failure, we recommend tying off the clamped site of the right bronchus with string.

## Resource availability

### Lead contact

Further information and requests for resources and reagents should be directed to and will be fulfilled by the lead contact, Masataka Sugimoto (msugimot@ncgg.go.jp).

### Materials availability

This study did not generate new unique reagents.

ARF-DTR mouse is available upon request to read contact.

## Data Availability

This study did not generate/analyze [datasets/code].
